# Intramolecular C-H···O Hydrogen Bonding in 1,4-Dihydropyridine Derivatives

**DOI:** 10.3390/molecules16098041

**Published:** 2011-09-19

**Authors:** Marina Petrova, Ruslan Muhamadejev, Brigita Vigante, Brigita Cekavicus, Aiva Plotniece, Gunars Duburs, Edvards Liepinsh

**Affiliations:** Latvian Institute of Organic Synthesis Aizkraukles 21 Street, Riga LV-1006, Latvia; Email: muhamadejev@osi.lv (R.M.); vigante@osi.lv (B.V.); arkady@osi.lv (B.C.); aiva@osi.lv (A.P.); gduburs@osi.lv (G.D.); edv@osi.lv (E.L.)

**Keywords:** 1,4-dihydropyridines, bromination, nucleophilic substitution, hydrogen bond, NMR spectra, quantum chemical calculations

## Abstract

The diastereotopy of the methylene protons at positions 2 and 6 in 1,4-dihydropiridine derivatives with various substituents has been investigated. NMR spectroscopy and quantum chemistry calculations show that the CH···O intramolecular hydrogen bond is one of the factors amplifying the chemical shift differences in the ^1^H-NMR spectra.

## 1. Introduction

The 1,4-dihydropyridine (1,4-DHP) scaffold is a common component of pharmacologically active molecules which possess a variety of biological activities [1]. Several 1,4-DHP derivatives play an important role in regulating the decrease or increase of the penetration of calcium ions through membranes into cells. Substituents at the 2,6-, 3,5-, 4- and 1- positions of 1,4-DHP derivatives influence their structure-activity relationships, so structural and conformational investigations of these systems are important to gain insight into the mechanisms of their physiological action [2]. 

## 2. Results and Discussion

In recent years many studies attempting to modify the 2,6-methyl groups of 1,4-DHP by introducing various substituents have been performed [3,4,5,6]. On the other hand cationic amphiphilic 1,4-DHP derivatives have gained significance as useful transport molecules for the delivery of nucleotides into target cells [4,7].

Our attention was drawn to the fact that the CH_2_X protons of substituents in positions 2 and 6 of symmetrically substituted 1,4-dihydropyridine rings (compounds **1-3**, **5**, Table 1) become diastereotopic in the presence of different substituents at position 4, thereby, providing an AB system in the corresponding ^1^H-NMR spectra. The extent of the observed non-equivalence of the methylene protons should be influenced by the spatial conformation of side chains in the molecule and/or the anisotropy of the substituents.

**Table 1:** Characterization of compounds **1–5**. 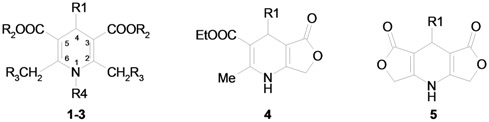

Comp.	R^1^	R^2^	R^3^	R^4^	Yield,%	Reference
**1a**	Me	Et	Br	H	59	
**1b**	Ph	Et	Br	Me	88	
**1c**	PhCF_3_-o	Et	Br	H	76	
**1d**	PhOCHF_2_-o	(CH_2_)_2_OC_3_H_7_-*n*	Br	H	61	
**1e**	COOMe	Et	Cl	H	87	
**1f**	Ph	Et	Br	H	47	[3]
**1g**	PhOCHF_2_-o	Me	Br	H	86	[3]
**1h**	Ph	C_12_H_25_	Br	H	72	[5]
**1i**	PhOCHF_2_-o	Me	Br	Me	89	
**2a**	Me	Et	Py^+^Br^−^	H	63	
**2b**	Ph	Et	Py^+^Br^−^	Me	66	
**2c**	PhCF_3_-o	Et	Py^+^Br^−^	H	93	
**2d**	PhOCHF_2_-o	(CH_2_)_2_OC_3_H_7_-*n*	Py^+^Br^−^	H	74	
**2e**	COOMe	Et	Py^+^Cl^−^	H	91	
**2f**	Ph	Et	Py^+^Br^−^	H	63	[6]
**2g**	PhOCHF_2_-o	Me	Py^+^Br^−^	H	64	[4]
**2h**	PhOCHF_2_-o	Me	Py^+^Br^−^	Me	84	
**3a**	Ph	C_10_H_21_	Py^+^Br^−^	H	63	[4]
**3b**	Ph	C_12_H_25_	Py^+^Br^−^	H	82	[4]
**3c**	Ph	C_14_H_29_	Py^+^Br^−^	H	35	[4]
**3d**	Ph	C_16_H_33_	Py^+^Br^−^	H	33	[7]
**3e**	Ph	C_12_H_25_	Py^+^Br^−^	Me	38	[7]
**4a**	Ph				20	[10]
**4f**	PhOCHF_2_-o				33	[11]
**5a**	Ph				50	[12]
**5f**	PhOCHF_2_-o				60	[13]

**Scheme 1:**
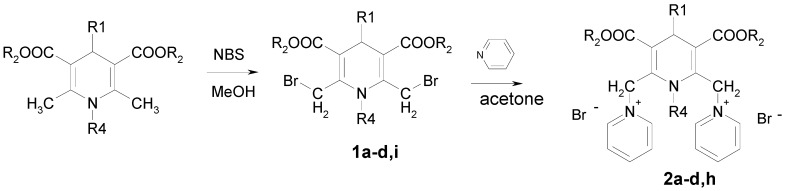
Synthesis of compounds **1** and **2**.

The synthetic pathway for obtaining 1,4-dihydropyridine derivatives bearing substituents on the 2,6-methylene groups (Table 1) involved Hantzsch synthesis, followed by bromination of the methyl groups with NBS and nucleophilic substitution of bromine by pyridine giving the target compounds **1a–d**, **i** and **2a–d**, **h** (Scheme 1).

**Scheme 2:**
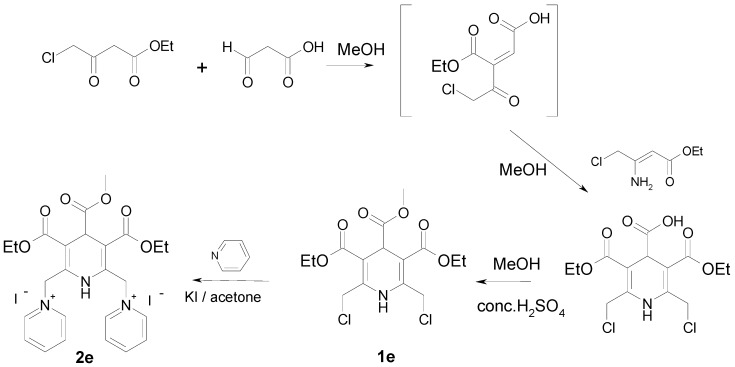
Synthesis of compounds **1e**, **2e**.

A different approach was elaborated for the synthesis of 4-methoxycarbonyl derivative **1e **(Scheme 2). Thus, the condensation of ethyl 4-chloroacetoacetate with glyoxylic acid monohydrate in methanol at rt in the presence of piperidine/acetate as catalyst provided (*E,Z*)-2-(2-chloroacetyl)-but-2-enedioic acid 1-ethyl ester, which was used without isolation in the next reaction with ethyl 3-amino-4-chlorobut-2-enoate. The obtained 3,5-diethyl 2,6-bis(chloromethyl)-1,4-dihydropyridine-3,4,5-tri-carboxylate was esterified with methanol using conc. sulfuric acid as catalyst to afford compound **1e**. The target compound **2e** was successfully obtained *via *nucleophilic substitution of chlorine by pyridine in the presence of potassium iodide in dry acetone.

Compounds **1f–h**, **2f–g**, **3a–e**, **4a,f** and **5a,f** were prepared according to the literature, as mentioned in Table 1. The 4-substituent in the molecule is located at a considerable distance from the 2- (6–) methylene group, and the experimentally observed difference in the ^1^H-NMR chemical shifts of the CH_2_ groups in cyclic lactones **4–5** does not exceed 0.08 ppm (Table 2); a similar value was observed in [8]. However, its potential anisotropic influence on the 2,6-CH_2_ methylene proton chemical shifts can’t be ignored. In monocyclic derivatives **1–3** the conformation of the 3,5-ethoxycarbonyl groups should be another important factor in the anisotropy. Indeed in these compounds the chemical shift difference for CH_2_X protons becomes very significant (Table 2), so we can assume that the difference in chemical shifts of CH_2_X groups in **1–3** induced by the anisotropy of the 4-substituent may also be influenced by the position of these protons relative to the 3,5-alkoxycarbonyl substituents. In order to study the substituent influence on the magnetic nonequivalence of the mentioned ^1^H-NMR signals, quantum chemical studies were carried out for some derivatives of 1,4-DHP (compounds **1–5**). The obtained data are presented in Table 3.

**Table 2:** ^1^H-NMR data of compounds **1–5**.

Comp.	Solvent	δNH	δH_B_	^1^J(C,H_B_)	δH_A_	^1^J(C,H_A_)	^2^J(H,H)
**1a**	CDCl_3_	6.35	4.89	159	4.51	157	11.5
	DMSO	9.44	4.69		4.40		9.6
**1b**	CDCl_3_		4.96		4.82		11.0
	DMSO		5.17		4.87		11.3
**1c**	CDCl_3_	6.49	4.81	159	4.66	157	11.6
	DMSO	9.67	4.61	158	4.56	157	9.9
**1d**	CDCl_3_	6.55	4.86		4.57		11.8
	DMSO	9.62	4.67		4.53		9.7
**1e**	CDCl_3_	7.37	5.19	160	4.74	158	14.6
	DMSO	9.78	4.93	158	4.48	156	11.1
**1f**	CDCl_3_	6.45	4.91	159	4.63	156.4	11.8
	DMSO	9.58	4.67	160	4.57	158.7	9.6
**1g**	CDCl_3_	6.56	4.86		4.62		11.3
	DMSO	9.65	4.62		4.59		9.7
**1h**	CDCl_3_	6.45	4.92	158	4.63	156	11.5
	DMSO	9.62	4.68	159	4.59	157	9.7
**1i**	CDCl_3_		4.91		4.84		11.0
	DMSO		5.18		4.85		11.4
**2a**	CDCl_3_	10.88	6.28		5.76		14.1
	DMSO	10.10	5.99		5.49		15.0
**2b**	CDCl_3_		6.82	152.1	6.59	146.3	15.6
	DMSO		6.45	152.2	5.77	146.2	16.0
**2c**	CDCl_3_	10.98	6.35	153.1	5.97	147.1	13.6
	DMSO	10.48	5.97	151.1	5.66	149.1	15.1
**2d**	CDCl_3_	10.80	6.16		6.11		14.1
	DMSO	10.15	5.90	150.1	5.62	149.2	14.9
**2e**	CDCl_3_	10.31	6.30		6.06		14.1
	DMSO	10.20	5.97		5.64		15.6
**2f**	CDCl_3_	10.92	6.38	150.6	5.92	146.5	13.6
	DMSO	10.34	6.08	151.9	5.63	146.4	15.3
**2g**	CDCl_3_	10.88	6.24		6.10		13.7
	DMSO	10.31	5.94		5.46		14.9
**2h**	CDCl_3_		6.84		6.62		15.8
	DMSO		6.39		5.77		16.2
**3a**	CDCl_3_	10.96	6.40	150.7	5.89	146.5	13.9
	DMSO	10.21	6.08	151.1	5.56	145.4	15.1
**3b**	CDCl_3_	10.95	6.40	151.4	5.89	146.3	13.9
	DMSO	10.12	6.07	152.4	5.53	146.3	15.0
**3c**	CDCl_3_	10.92	6.32	150.1	5.86	146.2	13.8
	DMSO	10.28	6.08	152.1	5.56	146.4	15.1
**3d**	CDCl_3_	10.96	6.40	148.3	5.89	146.2	13.9
	DMSO	10.12	6.07		5.53		15.2
**3e**	CDCl_3_		6.73	152.5	6.56	150.2	16.1
	DMSO		6.48	152.5	5.78	147.5	16.5
**4a**	CDCl_3_	8.30	4.52	and	4.47		16.7
	DMSO	9.74	4.84	and	4.74		16.5
**4f**	CDCl_3_	6.42	4.69	and	4.65		16.4
**5a**	DMSO	9.91	4.72	and	4.65		16.0
**5f**	DMSO	9.97	4.64	and	4.60		16.2

**Table 3:** Comparison of H-bond parameters in model compounds **1f**, **1b** and **2b**, **2f** and **3b**.

Compound	C2(CH···O)	C6(CH···O)	C2 pyr(CH···O)	C6 pyr(CH···O)
**1f**	2.508 Å,	2.511 Å,	-	-
	(86.6°)	(86.5°)		
	2.376 Å,	2.374 Å,		
	(93.6°)	(93.7°)		
**1b**	2.032 Å,	2.073 Å,	-	-
	(131.5°)	(126.0°)		
**2f**	2.329 Å,	2.168 Å,	2.629 Å,	2.152 Å,
	(99.6°)	(117.7°)	(89.7°)	(137.3°)
**2b**	2.254 Å,	2.198 Å,	2.544 Å,	2.913 Å,
	(103.9°)	(107.7°)	(94.0°)	(87.5°)
**3b**	2.341 Å,	2.366 Å,	2.591 Å,	2.849 Å,
	(98.914°)	(96.979°)	(90.362°)	(82.121°)

Signals of the AB protons of the 2,6-methylene groups for compounds **1–5** are easily distinguishable in the ^1^H-NMR spectra by their 11–15 Hz geminal ^2^*J* coupling constants. The values of the constants for compounds **1** are less than for compounds **2** and **3**. Neighbouring π-bonds of the pyridines in **2**, **3** usually result in an increase of the absolute value of the constants ^2^*J*. 

According to literature data [9], increasing the polarity of the medium usually slightly increases the negative contribution to the geminal coupling, and if its sign is negative, the absolute value increases. Such effect is observed in the N-methyl-substituted derivatives **1b**, **1i** and in compounds **2**, **3** where |^2^*J*_cdcl3_| < |^2^*J*_dmso_|. However, for compounds **1a** and **1c–h**, contrary to the literature data, the ^2^*J* values decrease by about 2 Hz on going from CDCl_3_ to DMSO solution. The unusual behaviour of the geminal couplings for compounds **1a**, **1c–h** may be explained by the variation of the dihedral angle between the methylene group protons and 1,4-DHP cycle in distribution of rotamers around the C_2,6_-CH_2_X bond. 

**Figure 1:**
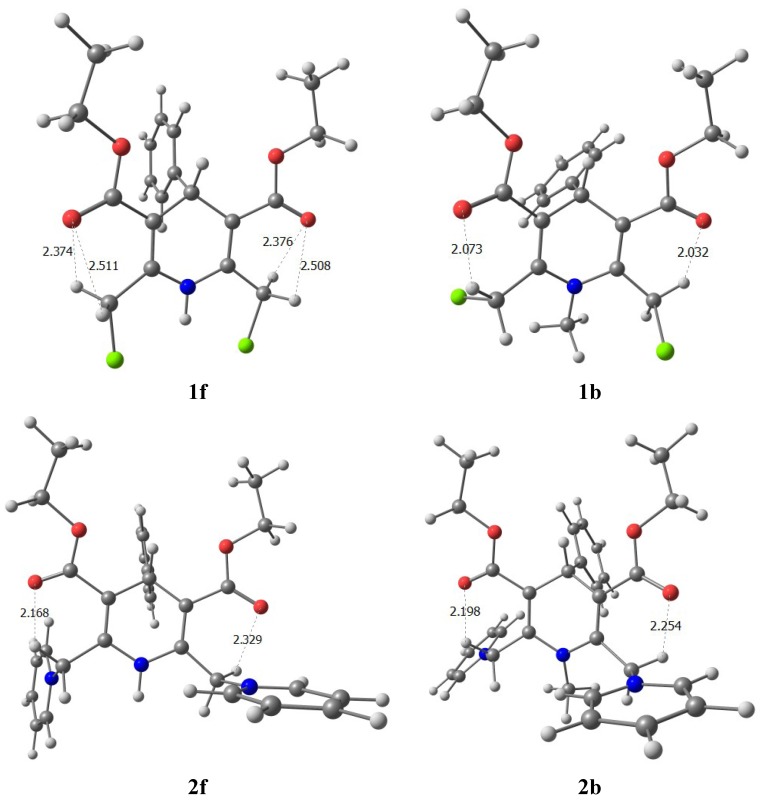
The calculated optimal conformations in compounds **1f**, **1b** and **2f**, **2b**.

Formation of intermolecular hydrogen bonds between the NH group and solvent DMSO moves the resonance signal of NH proton to a higher-frequency field ~3 ppm (Table 2). Contrary in compounds **2a**,**c–g** and **3a–d**, the resonance signal of the NH proton moves to a lower-frequency field ~0.5–1.1 ppm on going from CDCl_3_ to DMSO. The reason for this anomalous behaviour of the NH proton could be that in CDCl_3_ solutions of these compounds the CH_2_Py substituent is strongly oriented and NH proton is located in a zone of negative anisotropy of the pyridinium ring. This is confirmed by calculations (Figure 1). 

**Figure 2:**
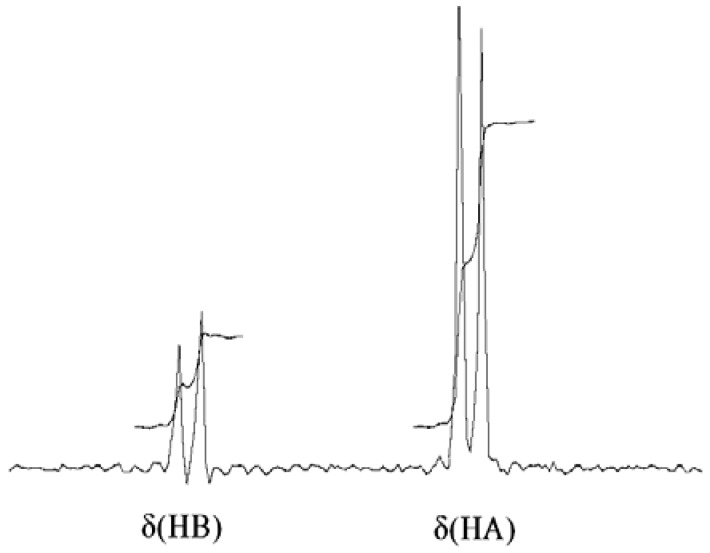
Slice of ^1^H-NMR NOESY spectrum for compound **3b**. More intense NOE is registered between NH and H_A_, showing preferable orientation toward the NH.

Analysis of NOEs in the ^1^H NOESY spectra of compounds **1**–**3** allows us to determine the relative position of the AB protons for the methylene group—the NOE from NH to the more shielded proton H_A_ is twice as intense as the H_B_ (Figure 2). This means that the H_A_ proton resonating in a lower-frequency field is located closer to the NH proton by about 0.3 angstroms.

Such an arrangement of CH_2_ protons leads to an intramolecular contact between the hydrogen atom H_B_ and the carboxyl group at the C3 carbon, leading to the formation of a CH_B_**···**O=C hydrogen bond. It is known that protons involved in hydrogen bonds are less shielded, as compared to free ones [14]. Short interactions of C-H**···**O play an important role in biology and have been observed in many cases previously [15].

**Figure 3:**
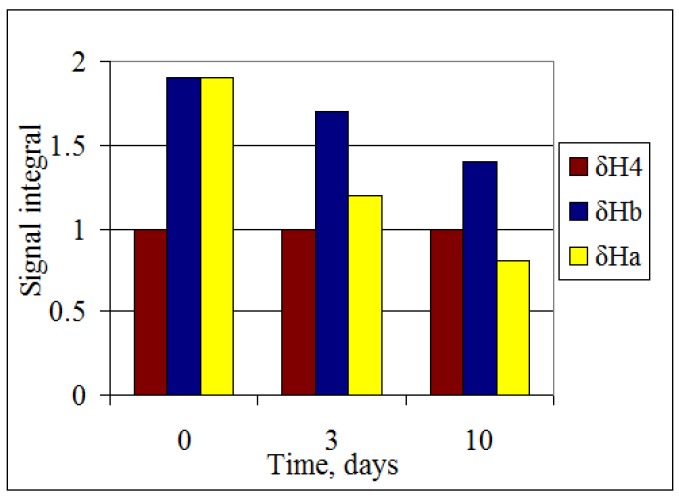
^1^H-NMR spectral data of D_2_O solutions of compound **3b** at 25 °C.

Additional confirmation for the existence of C-H**···**O hydrogen bonding in compounds **1–3** comes from the different deuteration rates of the 2(6)-CH_2_ protons (Figure 3). In D_2_O solution the intensity of the resonance signal of the more shielded proton H_A_ of compound **3b** decreases faster than for the more deshielded H_B_. This is because the hydrogen-bonded protons are usually less influenced by the intermolecular H-D exchange. This fact confirms the formation of a hydrogen bond between H_B_ methylene proton and the carboxyl group.

The measured coupling constants ^1^*J*(^13^C, ^1^H) for each of the methylene protons also differ ^1^*J*(^13^C, ^1^H_A_) < ^1^*J*(^13^C, ^1^H_B_) (see Table 2). The smaller ^1^*J*(^13^C, ^1^H) value corresponds to the more shielded proton H_A_. This is consistent with the literature data [16], which states that if the CH-proton is involved in a hydrogen bond, the ^1^*J*(^13^C, ^1^H) value increases.

^1^H-^13^C-HMBC spectra reveal a more intensive magnetization transfer from the low field H_B_ proton to ^13^C_3_ carbon as compared to H_A_. It is known that the intensities of cross peaks in the HMBC spectra are proportional to the value of the vicinal coupling constant ^3^*J*(^13^C, ^1^H), normally being ^3^*J*(C,H)_trans_ > ^3^*J(*C,H)_cis_ [17]. The delay value used to generate long-range spin-spin interaction between ^13^C-^1^H in HMBC spectrum was 8 Hz. The higher intensity of H_A_ cross peak to the carbon C_3_ (110.1 ppm) points to its trans-orientation relative to C_3_. Therefore, the orientation of the less shielded proton H_B_ favours its participation in the CH**···**O hydrogen bond.

Thus, on the basis of these data, we can unambiguously claim that the intramolecular CH**···**O hydrogen bond is one of the reasons for the non-equivalence of the methylene protons in these systems. In compounds **1** and **2** the difference in the chemical shifts of the AB methylene protons (Δδ_AB_ = δ_B_ − δ_A_) vary in the range 0–0.5 ppm, rather randomly, depending on the nature of substituents and the solvent used. In compounds **3a–d**, with bulk aliphatic substituents at C_3,5_, the difference is relatively constant and is equal to ~0.5 ppm (Table 2). To get more insight for the results obtained by NMR quantum-chemical calculations were carried out.

The results obtained reveal that for compounds **2b**, **2f** the energy minima correspond to the conformations with s-*cis-s-cis* carboxyl group orientation relative to the double bond in the dihydropyridine cycle, indicating the dominant stabilizing effect of intramolecular hydrogen bond on the conformation of molecules **2b**, **2f** the same as in case of **1b**, **1f**. Structures with s-*cis-s-trans* and *s-trans-s-trans* carboxyl groups orientation are with higher energy, consequently the stabilizing effect in solutions has to be attributed to the hydrogen bonds C-H**···**O. The calculated equilibrium conformations and the internuclear distances R (CH**···**O) also reveal the formation of a hydrogen bond (Table 3).

In description of hydrogen bonds of the CH**···**O type geometric characteristics are often used. In our case, the calculated parameters of intramolecular hydrogen bonds for compounds **1b**, **1f**, **2b**, **2f** and **3b**, presented in Table 3, are consistent with the X-ray diffraction and NMR spectral data. According to calculations, the distance d(CH**···**O) decreases, and the angle (C-H**···**O) increases on going from **1f**, **1b** to **3b**, indicating weakening of the hydrogen bond between the methylene H_B_ proton and oxygen of the carboxyl group. At the same time, in the most energetically favourable conformations of **2b**, **2f** and **3b** there exist additional H-bonds between the pyridine *ortho* protons and the oxygen atoms of the carboxyl group (see Table 3). These additional H-bonds also stabilize the structure of the molecule. In conformations with s-*cis-s-trans* and s-*trans-s-trans* orientation of the carboxyl groups relative to the double bonds of DHP cycle there is a possibility to form H-bonds with a length of about 2.400 Å between protons in position 4 and the ethoxycarbonyl oxygen. The latter contact is much weaker than the ones with protons of the methylene groups and does not contribute significantly to the conformation of the molecule.

In summary, some novel derivatives of 1,4-dihydropyridine bearing substituents at 2,6-methylene groups were synthesized. It was shown that one of the reasons for the diastereotopy of the methylene protons at positions 2 and 6 of symmetrically substituted 1,4-dihydropyridine rings is the formation of an intramolecular hydrogen bond of the CH**···**O type, which was confirmed by NMR spectroscopy and quantum-chemistry calculations.

## 3. Experimental

### 3.1. Chemicals

All reagents were purchased from Aldrich, Acros, Fluka or Merck and used without further purification. TLC was performed on 20 × 20 cm silica gel TLC-PET F254 foils (Fluka). Melting points were determined on an SRS OptiMelt apparatus (Stanford Research Systems, Sunnyvale, CA, USA). Elemental analyses were performed on an EA 1106 (Carlo Erba Instruments, Milan, Italy). Compounds were recrystallized from methanol, acetone or purified by column chromatography.

### 3.2. NMR Experiments (Table 2)

The one-dimensional ^1^H- and ^13^C- and two dimensional ^1^H-^1^H NOESY, ^13^C-^1^H HMBC, ^13^C-^1^H HSQC of compounds **1–5** were recorded on a Varian-Mercury 400 MHz BB instrument. The mixing time in the 2D-NOESY spectra was 800 ms. The ^13^C-HMBC were recorded with the evolution time of 62.5 s delay for the generation of long-range correlations. For all two dimensional spectra a 4,096 × 1,024 data matrix was used. To improve the signal-noise ratio, the data matrix before Fourier transformation was zero-filled twice and multiplied by a cosine function. The chemical shifts of the hydrogen and carbon atoms are presented in ppm and referred to the residual signals of the solvent a 7.25 (^1^H) and 77.0 ppm (^13^C) ppm, respectively. Multiplicities are abbreviated as: s, singlet; d, doublet; t, triplet; q, quartet; m, multiplet; br, broad. The coupling constants are expressed in Hertz.

### 3.3. Synthesis

*General method for the synthesis of 2,6-di(bromomethyl)-3,5-bis(alkoxycarbonyl)-4-aryl(4-alkyl)-(or N-methyl)-1,4-dihydropyridines ***1a–d**,**i**: To a solution of the appropriate 2,6-dimethyl-3,5-bis(alkoxycarbonyl)-4-aryl(4-alkyl)-1,4-dihydro-pyridine (5 mmol) in methanol (50 mL) NBS (10 mmol) was added portionwise at ambient temperature. The reaction mixture was stirred at rt for 24 h. The pale yellow precipitate was filtered and washed with water. The precipitate was crystallized from ethanol giving **1a–d**,**i**.

*2,6-Di(chloromethyl)-3,5-bis(ethoxycarbonyl)-4-methoxycarbonyl-1,4-dihydropyridine* (**1e**). A mixture of ethyl 4-chloroacetoacetate (2.72 mL, 20 mmol) and glyoxylic acid monohydrate (1.74 g, 19 mmol) in methanol (40 mL) was stirred at rt for 72 h in the presence of piperidine/acetate as catalyst. Then ethyl (*E,Z*)-3-amino-4-chlorobut-2-enoate (3.27 g, 20 mmol) was added and the combined mixture was stirred at rt additional 72 h. The solvent was removed under vacuum and the residue was washed with water. The obtained yellow oil was crystallized from methanol yielding 3,5-diethyl 2,6-bis-(chloromethyl)-1,4-dihydropyridine-3,4,5-tricarboxylate (4.98 g, 68%); m.p.164–166 °C; elemental analysis calcd (%) for C_14_H_17_Cl_2_NO_6_: C 45.92, H 4,68, N 3.82; found: C 45.95, H 4.58, N 3.72. To a solution of 3,5-diethyl 2,6-bis(chloromethyl)-1,4-dihydropyridine-3,4,5-tricarboxylate (1.83 g, 5 mmol) in methanol (30 mL) conc. sulfuric acid (0.1 mL) was added and the reaction mixture was refluxed for 2 h. The solvent was removed under vacuum and the residual crude product was triturated with water. After cooling the precipitate was filtered off and crystallized from methanol giving compound **1e** (1.65 g, 87%); m.p. 99–101 °C; p elemental analysis calcd. (%) for C_15_H_19_Cl_2_NO_6_: C 47.38, H 5.04, N 3.68; found: C 47.41; H 4.95; N 3.68.

*General method for the synthesis of 1,1`-{[3,5-bis(alkoxycarbonyl)(3,5-bis(2-propoxyethoxycarbonyl)-4-aryl(4-alkyl) (or N-methyl)-1,4-dihydropyridine-2,6-diyl] dimethylene} bispyridinium dibromides ***2a–d,h**: To a solution of compounds **1a–d**,**i** (2 mmol) in dry acetone (20 mL) pyridine (0.20 mL, 2.2 mmol) was added and the reaction mixture was stirred at rt for 24 h. After cooling the precipitate was filtered off, washed with dry acetone and crystallized from ethanol giving compounds **2a–d**,**h** as pale yellow powders.

*1,1`-{[3,5-Bis(ethoxycarbonyl)-4-methoxycarbonyl-1,4-dihydropyridine-2,6-diyl]dimethylene} bis-pyridinium diiodide* (**2e**). To a solution of compound **1e** (0.38 g, 1 mmol) in dry acetone (4 mL) pyridine (0.18 mL, 2 mmol) and potassium iodide (0.33 g, 2 mmol) were added and the mixture was stirred at rt for 48 h. The mixture was diluted with acetone (20 mL), and the potassium chloride formed was filtered off. Acetone was removed under vacuum, and the residue was crystallized from methanol yielding compound **2e** as a pale yellow powder, (0.65 g, 91%); m.p.137–139 °C; elemental analysis calcd (%) for C_25_H_29_I_2_N_3_O_6_: C 41.63; H 4.05; N 5.83; found: C 41.49; H 3.98; N 5.65.

### 3.4. Computional Methods

The Firefly (7.1.G) software package [18] was used for calculation/optimisation of structures/structures’ geometry. The structures of **1d**, **1e**, **1d** and **2a** were optimised using a combined RHF/DFT (b3lyp) method and 6-31g basis set. In the calculation of compounds **1** and **2** the basis set was supplemented by two polarization functions (**) and a diffuse (++) - 6–31g ++ ** (hybrid DFT/RHF B3LYP/6-31++G**). For **3b** the same method and theory level was used, only simplified by using one polarization function and one diffuse - (hybrid DFT/RHF B3LYP/6-31+G*).

## 4. Conclusions

Some novel derivatives of 1,4-dihydropyridine were synthesized. Due to the presence of a prochiral centre at C-4 in the heterocyclic ring magnetic non-equivalence of the diastereotopic methylene protons in positions 2 and 6 was observed. NMR spectroscopy and quantum-chemistry calculations confirmed that the formation of a CH···O intramolecular hydrogen bond amplifies the extent of the anisochrony of the methylene protons.

## Supplementary Materials

Supplementary File 1
