# Supplementary material for: Intramolecular C-H···O Hydrogen Bonding in 1,4-Dihydropyridine Derivatives
Source: Molecules. 2011 Sep 19;16(9):8041–52. doi: 10.3390/molecules16098041 (PMC6264772; doi:10.3390/molecules16098041)
Supplement: Supplementary File 1 [file molecules-16-08041-s001.pdf]

1. Experimental data of synthesised 1,4-dihydropyridines

2,6-Di(bromomethyl)-3,5-bis(ethoxycarbonyl)-4-methyl-1,4-dihydropyridine (1a). Yield 1.25g, 59%, mp 92-94 ^{0}C. Anal. calcd. for $C_{14}H_{19}Br_2NO_4$: C, 39.55; H, 4.50; N, 3.29; found: C, 39.91; H, 4.37; N, 3.27.

2,6-Di(bromomethyl)-3,5-bis(ethoxycarbonyl)-4-phenyl-N-methyl-1,4-dihydropyridine (1b). Yield 2.19 g, 88 %, mp 124-126 ^{0}C. Anal. calcd. for $C_{20}H_{23}Br_2NO_4$: C, 47.92; H, 4.62; N, 2.79; found: 48.38; H, 4.61; N, 2.77.

2,6-Di(bromomethyl)-3,5-bis(ethoxycarbonyl)-4-(2-trifluoromethylphenyl)-1,4-dihydropyridine (1c). Yield 2.10 g , 76%, mp 111-112 ^{0}C. Anal. calcd. for $C_{20}H_{20}Br_2F_3NO_4$: C, 43.27; H 3.63; N 2.52; found: C, 43.52; H, 3.40; N, 2.42.

2,6-Di(bromomethyl)-3,5-bis(2-propoxyetoxycarbonyl)-4-(2-difluoromethoxyphenyl)-1,4-dihydropyridine (1d). Yield 2.1 g, 61%, mp 102-105 ^{0}C. Anal. calcd. for $C_{26}H_{33}Br_2F_2NO_7$: C, 46.65; H, 4.97; N, 2.09; found: 46.83; H, 4.91; N, 2.05.

1,1`-{[3,5-Bis(ethoxycarbonyl)-4-methyl-1,4-dihydropyridine-2,6-diyl]dimethylene}bispyridinium dibromide (2 a). Yield 0.73 g, 63%, mp 195-197 ^{0}C. MS(+ESI) *m/z* (relative intensity) 423 [(M-2Br]$^{+}$, 20). Anal. calcd. for $C_{24}H_{29}Br_2N_3O_4$: C, 49.42; H, 5.01; N, 7.20; found: C, 49.80; H, 4.83; N, 7.00.

1,1`-{[3,5-Bis(ethoxycarbonyl)-4-phenyl-N-methyl-1,4-dihydropyridine-2,6-diyl]dimethylene}bispyridinium dibromide (2 b). Yield 0.86 g, 66%, mp 196-200 ^{0}C. MS(+ESI) *m/z* (relative intensity) 499 [(M-2Br]$^{+}$, 10). Anal.calcd. for $C_{30}H_{33}Br_2N_3O_4$: C, 54.64; H, 5.04; N, 6.37; found: C, 54.92; H, 4.80; N, 6.08.

1,1`-{[3,5-Bis(ethoxycarbonyl)-4-(2-trifluoromethylphenyl)-1,4-dihydropyridine-2,6-diyl]dimethylene}bispyridinium dibromide (2 c). Yield 1.32 g, 93%, mp 204-205 ^{0}C. MS(+ESI) *m/z* (relative intensity) 553 [(M-2Br]$^{+}$, 15). Anal. calcd. for $C_{30}H_{30}Br_2F_3N_3O_4$: C, 50.51; H, 4.24; N, 5.89; found: C, 50.23; H, 4.15; N, 5.73.

1,1`-{[3,5-Bis(2-propoxyethoxycarbonyl)-4-(2-difluoromethoxyphenyl)-1,4-dihydropyridine-2,6-diyl]dimethylene}bispyridinium dibromide (2d). Yield 1.23 g, 74%, mp 174 ^{0}C. MS(+ESI) *m/z*

(relative intensity) 669 [(M-2Br]$^+$, 31).Anal. calcd. for $C_{36}H_{43}Br_2F_2N_3O_7$: C, 52.25; H 5.23; N 5.08; found: C, 52.24; H, 5.17; N, 4.89.

1,1`-{[3,5-Bis(methoxycarbonyl)-4-(2-difluoromethoxyphenyl)-N-methyl-2,6-diyl]dimethylene}bispyridinium dibromide (2 h). Yield 1.15 g, 82%, mp 198-200 ^{0}C. MS(+ESI) *m/z* (relative intensity) 537 [(M-2Br]$^+$,25). Anal. calcd. For $C_{29}H_{29}Br_2F_2N_3O_5$: C, 49.95; H, 4.19; N, 6.03; found: C, 50.15; H, 4.30; N, 5.86.

2. Table of ^{1}H NMR spectral data.

Table of ^{1}H NMR spectral data

Comp.	Solvent	Residual ^{1}H NMR signals of compounds **1-5**
**1a**	$CDCl_3$	1.02(d, J=6,8Hz, 3H, CH_3) 1.32(t, J=7,2 Hz, 6H, $2CH_3$), 3.89(kv, J=6.8 Hz, 1H, CH), 4.22-4.29(m, 4H, $2CH_2$)
	DMSO	0.82(d, J=6,6Hz, 3H, CH_3), 1.22(t, J=7,1Hz, 6H, $2CH_3$), 3.71(kv, J=6.6Hz, 1H, CH), 4.10-4.19(m, 4H, $2CH_2$)
**1b**	$CDCl_3$	1.31(t, J=7,3Hz, 6H, $2CH_3$), 3.46(s,3H, NCH_3), 4.24(kv, J=7.3Hz, 4H, $2CH_2$), 5.23(s, 1H, CH), 7.16-7.23 (m, 5H, C_6H_5)
	DMSO	1.21(t, J=7,2Hz, 6H,$2CH_3$), 3.42(s,3H, CH_3), 4.14(kv, J=7.2Hz, 4H, $2CH_2$), 5.03(s, 1H, CH), 7.08-7.23 (m, 5H, C_6H_5)
**1c**	$CDCl_3$	1.20(t, J=7.1Hz, 6H, $2CH_3$), 4.03-4.21(m, 4H, $2CH_2$), 5.62 (kv, J=1Hz, 1H, CH), 7.29-7.51(m, 4H, C_6H_4)
	DMSO	1.10(t, J=7.1Hz, 6H, $2CH_3$), 3.94-4.06(m, 4H, $2CH_2$), 5.44(kv, J=1 Hz, 1H, CH), 7.37-7.56(m, 4H, C_6H_4)
**1d**	$CDCl_3$	0.88(t, J=6.8Hz, 6H, $2CH_3$), 1.55(m, 4H, $2CH_2$), 3.35(t, J=6.8Hz, 4H, $2CH_2$), 3.59 (m, 4H, $2CH_2$), 4.20(t, J=4.9Hz, 4H, $2CH_2$), 5.33(s, 1H, CH), 60(t, J=75Hz, 1H, CH) 7.01(d, J=8.4Hz, 1H, C_6H_4), 7.08(td, J=7.4; J=0.9Hz, 1H, C_6H_4), 7.17(td, J=7.6 Hz, J=0.9 Hz, C_6H_4), 7.37(dd, J=7.8 Hz, J=1.6 Hz, 1H, C_6H_4)
	DMSO	0.81(t, J=7.5Hz, 6H, $2CH_3$), 1.44(m, 4H, $2CH_2$), 3.28(t, J=6.8Hz, 4H, $2CH_2$), 3.52(t, J=4.7Hz, 4H, $2CH_2$), 4.06-4.12(m, 4H, $2CH_2$), 5.22(s, 1H, CH), 7.01(d, J=8.3Hz, 1H, C_6H_4), 7.04(t, J=74Hz, 1H, C_6H_4), 7.09(td, J=7.6 Hz, J=0.8 Hz, 1H, C_6H_4), 7.20(td, J=7.6 Hz, J=0.9 Hz,1H, C_6H_4), 7.26(dd, J=7.6 Hz, J=1.2 Hz, 1H, C_6H_4)
**1e**	$CDCl_3$	1.31(t, J=7.1Hz, 6H, $2CH_3$), 3.64(s,3H, CH_3), 4.20-4.30 (m, 4H, $2CH_2$), 4.97(s, 1H, CH)
	DMSO	1.21(t, J=7.1Hz, 6H, $2CH_3$), 3.51(s, 3H, CH_3), 4.11-4.21(m, 4H, $2CH_2$), 4.79(s, 1H, CH)
**1f**	$CDCl_3$	1.25(t, J=7Hz, 6H, $2CH_3$), 4.14(m, 4H, $2CH_2$), 5.02(s, 1H, CH), 7.17-7.27(m, 5H, C_6H_5)
	DMSO	1.15(t, J=7 Hz, 6H, $2CH_3$), 4.05(m, 4H, $2CH_2$), 4.89(s, 1H, CH), 7.12-7.24(m, 5H, C_6H_5)
**1g**	$CDCl_3$	3.66(s, 6H, $2CH_3$), 5.35(s, 1H, CH), 6.50 (t, J=75Hz, 1H, CH), 7.01(d, J=7.6 Hz, 1H, C_6H_4), 7.10(t, J=7.6Hz, 1H, C_6H_4), 7.19(t, J=7.6Hz, 1H, C_6H_4), 7.33(d, J=7.6Hz, 1H, C_6H_4)
	DMSO	3.55(s, 6H, $2CH_3$), 5.26(s, 1H, CH), 7.02(d, J=7.8Hz, 1H, C_6H_4), 7.10(td, J=7.5Hz, J=1.1Hz, 1H, C_6H_4), 7.12(t, J=74.3Hz, 1H, CH), 7.21(td, J=7.5Hz, J=1.8Hz, 1H, C_6H_4), 7.24(dd, J=7.5, J=1.8Hz, 1H, C_6H_4)
**1h**	$CDCl_3$	0.89(t, J=7Hz, 6H, $2CH_3$), 1.27(m, 36H, $18CH_2$), 1.61(t, J=7 Hz, 4H, $2CH_2$), 4.07(m, 4H, $2CH_2$), 5.02(s,1H), 7.16-7.28(m, 5H, C_6H_5)
	DMSO	0.83(t, J=7Hz, 6H, $2CH_3$), 1.23(m, 36H, $18CH_2$), 1.53(m, 4H, $2CH_2$), 3.98(t, J=6.1Hz, 4H, $2CH_2$), 4.91(s,1H), 7.10-7.21(m, 5H, C_6H_5)
**1i**	$CDCl_3$	3.49(s, 3H, NCH_3), 3.75 (s, 6H, $2CH_3$), 5.26(s, 1H, CH), 6.50(t, J=75.5 Hz, 1H, CHF_2), 7.01 (d, J=8.2Hz, 1H, C_6H_4), 7.07(td, J=7.8Hz, J=1.1Hz, 1H, C_6H_4), 7.11(dd, J=7.7Hz, J=1.8Hz, 1H, C_6H_4), 7.19(td, J=7.7Hz, J=1.8Hz, 1H, C_6H_4)
	DMSO	3.41(s, 3H, NCH_3), 3.66 (s, 6H, $2CH_3$), 5.35(s, 1H, CH), 7.0 (dd, J=7.8Hz; 1.3 Hz, 1H, C_6H_4), 7.06 (td, J=7.8Hz, J=1.3Hz, 1H, C_6H_4), 7.19(t, J=74 Hz, 1H, CHF_2), 7.23(m, 2H,C_6H_4)
**2a**	$CDCl_3$	1.16(d, J=6.5Hz, 3H, CH_3), 1.33(t, J=7.4Hz, 6H, $2CH_3$), 3.96(kv, J=6.5Hz,1H, CH), 4.21-4.30(m, 4H, $2CH_2$), 8.20(t, J=7.4Hz, 4H, $2C_6H_4N$), 8.69(t, J=7.4Hz, 2H, $2C_6H_4N$), 9.31(dd, J=7.4Hz, J=1.2Hz, 4H, $2C_6H_4N$).
	DMSO	1.03(d, J=6.5Hz, 3H, CH_3), 1.15(t, J=7.4Hz, 6H, $2CH_3$), 3.86(kv, J=6.5Hz,1H, CH), 4.09-4.19(m, 4H, $2CH_2$), 8.06(t, J=7.4Hz, 4H, $2C_6H_4N$), 8.55(t, J=7.5Hz, J=1.1Hz, 2H, $2C_6H_4N$), 8.91(dd, J=7.5Hz, J=1.2Hz, 4H, $2C_6H_4N$)
**2b**	$CDCl_3$	1.30(t, J=7.7Hz, 6H, $2CH_3$), 3.55(s, 3H, CH_3), 4.22 (kv, J=7.7Hz, 4H, $2CH_2$), 5.26(s, 1H, CH), 7.26-7.36 (m, 5H, C_6H_5), 8.10(t, J=7.2Hz, 4H, $2C_6H_4N$), 8.43(t, J=7.2Hz, 2H, $2C_6H_4N$), 9.39(d, J=7.2Hz, 4H, $2C_6H_4N$)

	DMSO	1.17(t, J=7.6Hz, 6H, $2CH_3$), 3.28(s, 3H, CH_3), 4.17 (kv, J=7.6Hz, 4H, $2CH_2$), 5.13(s, 1H, CH), 7.26-7.34 (m, 5H, C_6H_5), 8.02(t, J=7.1Hz, 4H, $2C_6H_4N$), 8.50(t, J=7.2Hz, 2H, $2C_6H_4N$), 8.84(d, J=7.2Hz, 4H, $2C_6H_4N$)
**2c**	$CDCl_3$	1,20(t, J=7.2Hz, 6H, $2CH_3$), 4.09-4.23 (m, 4H, $2CH_2$), 5.79(kv, J=2.1Hz, 1H, CH), 7.33(t, J=7.8 Hz, 1H, C_6H_4), 7.54(d, J=7.8Hz, 1H, C_6H_4), 7.59(d, J=7.8,Hz, 1H, C_6H_4), 7.69(t, J=7.8Hz, 4H, C_6H_4), 8.20(t, J=7.1Hz, 4H, $2C_6H_4N$), 8.55(t, J=7.1Hz, 2H, $2C_6H_4N$), 9.40(d, J=7.1Hz, 4H, $2C_6H_4N$)
	DMSO	1,02(t, J=7.2Hz, 6H, $2CH_3$), 3.92-4.06 (m, 4H, $2CH_2$), 5.58(kv, J=2.1Hz, 1H, CH), 7.42(t, J=7.6 Hz, 1H, C_6H_4), 7.57(d, J=7.6Hz, 1H, C_6H_4), 7.62(t, J=7.6,Hz, 1H, C_6H_4), 7.70(d, J=7.6Hz, H, C_6H_4), 8.13(t, J=7.4Hz, 4H, $2C_6H_4N$), 8.59(t, J=7.4, 2H, $2C_6H_4N$), 9.06(d, J=7.4Hz, 4H, $2C_6H_4N$)
**2d**	$CDCl_3$	0.84(t, J=7,8Hz, 6H, $2CH_3$), 1.49(m, 4H, $2CH_2$), 3.30(m, 4H, $2CH_2$), 3.52(m, 4H, $2CH_2$), 4.18(m, 4H, $2CH_2$), 5.33(s, 1H, CH), 6.91(t, J=64.7Hz, 1H, CHF_2), 7.03(t, J=7.8Hz, 1H, C_6H_4), 7.06(d, J=7.8, J=1.8Hz, 1H, C_6H_4), 7.19(td, J=7.8Hz, J= 1.8Hz, 1H, C_6H_4), 7.36(dd, J=7.8Hz, J=1.8Hz, 1H, C_6H_4), 8.19(t, J=7.8Hz, 4H, $2C_6H_4N$), 8.67(td, J=7.8Hz, J=1.2Hz, 2H, $2C_6H_4N$), 9.44(dd, J=7.8Hz, J=1.1 Hz, 4H, $2C_6H_4N$).
	DMSO	0.77(t, J=7,8Hz, 6H, $2CH_3$), 1.38(m, 4H, $2CH_2$), 3.20(t, J=6.8Hz, 4H, $2CH_2$), 3.44(m, 4H, $2CH_2$), 4.07(m, 4H, $2CH_2$), 5.32(s, 1H, CH), 7.04(d, J=7.6Hz, 1H, C_6H_4), 7.11(t, J=74.3Hz, 1H, CHF_2) 7.12(t, J=7.3Hz, 1H, C_6H_4), 7.28(t, J=7.8Hz, 1H, C_6H_4), 7.42(d, J=7.8Hz, 1H, C_6H_4), 8.11(t, J=7.7Hz, 4H, $2C_6H_4N$), 8.60(t, J=7.7Hz, 2H, $2C_6H_4N$), 9.03(d, J=7.2Hz, 4H, $2C_6H_4N$).
**2e**	$CDCl_3$	1.29(t, J=7.4Hz, 6H, $2CH_3$), 3.69(s, 3H, CH_3), 4.19-4.30 (m., 4H, $2CH_2$), 5.11 (s, 1H, CH), 8.17 (t, J=7.1Hz, 4H, $2C_6H_4N$), 8.60(t, J=7.1Hz, 2H, $2C_6H_4N$), 9.32(d, J=7.6Hz, 4H, $2C_6H_4N$)
	DMSO	1.13(t, J=7.4Hz, 6H, $2CH_3$), 3.58(s, 3H, CH_3), 4.09-4.19 (m., 4H, $2CH_2$), 4.91 (s, 1H, CH), 8.12 (t, J=7.2Hz, 4H, $2C_6H_4N$), 8.60(t, J=7.2Hz, 2H, $2C_6H_4N$), 8.89(d, J=7.6Hz, 4H, $2C_6H_4N$)
**2f**	$CDCl_3$	1,23(t, J=7.1Hz, 6H, $2CH_3$), 4.09(m, 4H, $2CH_2$), 5.10(s, 1H, CH), 7.21-7.28 (m, 5H, C_6H_5), 8.19(t, J=7.5Hz, 4H, $2C_6H_4N$), 8.60(tt, J=7.5Hz, J=1Hz,1Hz, 2H, $2C_6H_4N$), 9.36(d, J=7.5Hz, 4H, $2C_6H_4N$),
	DMSO	1,18(t, J=7.4Hz, 6H, $2CH_3$), 4.04(m, 4H, $2CH_2$), 5.01(s, 1H, CH), 7.18-7.28 (m, 5H, C_6H_5), 8.10(t, J=7.5Hz, 4H, $2C_6H_4N$), 8.58(tt, J=7.5, J=1.2Hz, 2H, $2C_6H_4N$), 8.99(dd, J=7.5,J=1.1Hz, 4H, $2C_6H_4N$)
**2g**	$CDCl_3$	3.68(s, 6H, $2CH_3$), 5.45(s, 1H, CH), 6.89(t, J=74.1Hz, 1H, CHF_2), 7.08 (d, J=7.6Hz, 1H, C_6H_4), 7.14 (t, J=7.6Hz, 1H, C_6H_4), 7.24(t, J=7.6Hz, 1H, C_6H_4), 7.34(dd, J=7.8Hz, J=1.7Hz, 1H, C_6H_4), 8.16(t, J=7.4Hz, 4H, $2C_6H_4N$), 8.56(t, J=7.5Hz, 2H, $2C_6H_4N$), 8.16(d, J=7.5Hz, 4H, $2C_6H_4N$)
	DMSO	3.31(s, 6H, $2CH_3$), 5.36(s, 1H, CH), 7.03(d, J=7.8, 1H, C_6H_4), 7.15 (t, J=7.8Hz, 1H, C_6H_4), 7.16 (t, J=74.2Hz, CHF_2), 7.27 (td, J=7.6Hz, 1H, C_6H_4), 7.41(dd, J=7.6Hz, J=1.2Hz, 1H, C_6H_4), 8.11(t, J=7.6Hz, 4H, $2C_6H_4N$), 8.59(t, J=7.6Hz, 2H, $2C_6H_4N$), 9.02(d, J=7.6Hz, 4H, $2C_6H_4N$)
**2h**	$CDCl_3$	3.53(s, 3H, NCH_3), 3.76(s, 6H, $2CH_3$), 6.68(t, J=74.2Hz, 1H, CHF_2), 5.53 (s, 1H), 7.09 (d, J=7.8Hz, 1H, C_6H_4), 7.26-7.33 (m, 3H,C_6H_4), 8.11(t, J=7.8Hz, 4H, $2C_6H_4N$), 8.49(t, J=7.8Hz, 2H, $2C_6H_4N$), 9.41 (d, J=7.8Hz, 4H, $2C_6H_4N$)
	DMSO	3.28(s, 3H, NCH_3), 3.76(s, 6H, $2CH_3$), 5.49 (s, 1H, CH), 7.17 (t, J=8.1Hz, 1H, C_6H_4), 7.20(d, J=8.1Hz, 1H, C_6H_4), 7.27(d, J=8.2Hz, 1H, C_6H_4), 7.28(t, J=73.6Hz, 1H, CHF_2), 7.34(t, J=8.2Hz, 1H, C_6H_4), 8.01(t, J=7.5Hz, 4H, $2C_6H_4N$), 8.50(t, J=7.6Hz, 2H, $2C_6H_4N$), 8.81(d, J=7.6Hz, 4H, $2C_6H_4N$)
**3a**	$CDCl_3$	0.89(t, J=6.5Hz, 6H, $2CH_3$), 1.25(m, 28H, $14CH_2$), 1.61(m, 4H, $2CH_2$), 4.07(t, J=6.5Hz, 4H, $2CH_2$), 5.09(s, 1H, CH), 7.22-7.29(m, 5H, C_6H_5), 8.19(t, J=7.5Hz, 4H, $2C_6H_4N$), 8.60(t, J=7.5Hz 2H, $2C_6H_4N$), 9.34(d, J=7.2, 4H, $2C_6H_4N$)
	DMSO	0.84(t, J=6.9Hz, 6H, $2CH_3$), 1.20(m, 28H, $14CH_2$), 1.49(m, 4H, $2CH_2$), 3.98(t, J=6.5Hz, 4H, $2CH_2$), 5.00(s, 1H, CH), 7.19-7.27(m, 5H, C_6H_5), 8.08(t, J=7.7Hz, 4H, $2C_6H_4N$), 8.57(t, J=7.7Hz, 2H, $2C_6H_4N$), 8.94(d, J=7.2Hz, 4H, $2C_6H_4N$)
**3b**	$CDCl_3$	0.88(t, J=6.9Hz, 6H, $2CH_3$), 1.26(m, 36H, 18CH2), 1.60(m, 4H, $2CH_2$), 4.07(t, J=7.1Hz, 4H, $2CH_2$), 5.08(s, 1H, CH), 7.20-7.29(m, 5H, C_6H_5), 8.20(t, J=7.3Hz, 4H, $2C_6H_4N$), 8.61(t, J=7.3Hz, 2H, $2C_6H_4N$), 9.34(d, J=7.3, 4H, $2C_6H_4N$)

	DMSO	0.84(t, *J*=6.9Hz, 6H, 2CH_3), 1.23(m, 36H, 18CH_2), 1.59(m, 4H, 2CH_2), 3.98(t, *J*=7.1Hz, 4H, 2CH_2), 5.01(s, 1H, CH), 7.18-7.28(m, 5H, C_6H_5), 8.07(t, *J*=7.2Hz, 4H, 2C_6H_4N), 8.56(t, *J*=7.2Hz, 2H, 2C_6H_4N), 8.92(d, *J*=7.2, 4H, 2C_6H_4N)
**3c**	$CDCl_3$	0.82(t, *J*=6.9Hz, 6H, 2CH_3), 1.22(m, 44H, 22CH_2), 1.54(m, 4H, 2CH_2), 4.01(t, *J*=7.1Hz, 4H, 2CH_2), 5.02(s, 1H, CH), 7.14-7.23(m, 5H, C_6H_5), 8.14(t, *J*=7.6Hz, 4H, 2C_6H_4N), 8.57(t, *J*=7.6Hz, 2H, 2C_6H_4N), 9.28(d, *J*=7.6, 4H, 2C_6H_4N)
	DMSO	0.83(t, *J*=6.9Hz, 6H, 2CH_3), 1.21(m, 44H, 22CH_2), 1.49(m, 4H, 2CH_2), 3.97(t, *J*=6.9Hz, 4H, 2CH_2), 5.01(s, 1H, CH), 7.17-7.28(m, 5H, C_6H_5), 8.08(t, *J*=7.5Hz, 4H, 2C_6H_4N), 8.57(t, *J*=7.5Hz, 2H, 2C_6H_4N), 8.95(d, *J*=7.5Hz, 4H, 2C_6H_4N)
**3d**	$CDCl_3$	0.88(t, *J*=7.2Hz, 6H, 2CH_3), 1.24(m, 52H, 26CH_2), 1.62(m, 4H, 2CH_2), 4.07(t, *J*=6.8Hz, 4H, 2CH_2), 5.09(s, 1H, CH), 7.22-7.29(m, 5H, C_6H_5), 8.19(t, *J*=7.1Hz, 4H, 2C_6H_4N), 8.59(t, *J*=7.1Hz, 2H, 2C_6H_4N), 9.34(d, *J*=7.1Hz, 4H, 2C_6H_4N)
	DMSO	0.83(t, *J*=7.2Hz, 6H, 2CH_3), 1.22(m, 52H, 26CH_2), 1.49(m, 4H, 2CH_2), 3.98(t, *J*=6.1Hz, 4H, 2CH_2), 5.01(s, 1H, CH), 7.19-7.27(m, 5H, C_6H_5), 8.07(t, *J*=7.3Hz, 4H, 2C_6H_4N), 8.56(t, *J*=7.3Hz, 2H, 2C_6H_4N), 8.56(d, *J*=7.3Hz 4H, 2C_6H_4N)
**3e**	$CDCl_3$	0.88(t, *J*=7.1Hz, 6H, 2CH_3), 1.28(m, 36H, 18CH_2), 1.65(m, 4H, 2CH_2), 3.61(s, 3H, NCH_3), 4.14(t, *J*=6.7Hz, 4H, 2CH_2), 5.25(s, 1H, CH), 7.25-7.36(m, 5H, C_6H_5), 8.16(t, *J*=7.5Hz, 4H, 2C_6H_4N), 8.49(t, *J*=7.5Hz, 2H, 2C_6H_4N), 9.38(d, *J*=7.5, 4H, 2C_6H_4N)
	DMSO	0.84(t, *J*=7.1Hz, 6H, 2CH_3), 1.25(m, 36H, 18CH_2), 1.57(m, 4H, 2CH_2), 3.12(s, 3H. NCH_3), 4.10(t, *J*=6.2Hz, 4H, 2CH_2), 5.15(s, 1H, CH), 7.18-7.31(m, 5H, C_6H_5), 8.02(t, *J*=7.2Hz, 4H, 2C_6H_4N), 8.50(t, *J*=7.2Hz, 2H, 2C_6H_4N), 8.86(d, *J*=7.2Hz, 4H, 2C_6H_4N)
**4a**	$CDCl_3$	1.09(t, *J*=7.3Hz, 3H, CH_3), 2.35(s, 3H, CH_3), 4.01(kv, *J*=7.2Hz, 2H, CH_2), 4.87 (s, 1H, CH), 7.18-7.27(m, 5H, C_6H_5)
	DMSO	1.01(t, *J*=7.5Hz, 3H, CH_3), 2.35(s, 3H, CH_3), 3.90(m, 2H, CH_2), 4.68 (s, 1H, CH), 7.12-7.25(m, 5H, C_6H_5)
**4f**	$CDCl_3$	2.32(s, 3H, CH_3), 3.48(s, 3H, CH_3), 5.13(s, 1H, CH), 6.50 and 6.61(dd, *J*=72Hz, *J*=75Hz, 1H, CHF_2), 6.99(d, *J*=7.8Hz, 1H, C_6H_4), 7.07(td, *J*=7.6Hz, *J*=1.6, 1H, C_6H_4), 7.13(td, *J*=7.6Hz, *J*=1.6Hz, 1H, C_6H_4), 7.22(dd, *J*=7.6Hz, *J*=1.6Hz, 1H, C_6H_4)
	DMSO	2.37(s, 3H, CH_3), 3.40(s, 3H, CH_3), 5.09(s,1H, CH), 6.57(dd, *J*=72Hz, *J*=74Hz, 1H, CHF_2), 6.93(dt, *J*=7.5Hz, *J*=1.6Hz, 1H, C_6H_4), 7.02(td, *J*=7.5Hz, *J*=1.6Hz, 1H, C_6H_4), 7.05(td, *J*=7.5Hz, *J*=1.6Hz, 1H, C_6H_4), 7.17(dd, *J*=7.5Hz, *J*=1.6Hz, 1H, C_6H_4)
**5a**	DMSO	4.67(s,1H, CH), 7.08-7.21(m, 5H, C_6H_5),
**5f**	DMSO	4.92(s,1H, CH), 6.54(t, *J*=75,2Hz, 1H), 6.93(d, *J*=7.5Hz, 1H, C_6H_4), 7.03(td, *J*=7.5Hz, *J*=1.9Hz, 1H, C_6H_4), 7.08(td, *J*=7.5Hz, *J*=1.9Hz, 1H, C_6H_4), 7.14(dd, *J*=7.5Hz, *J*=1.9Hz, 1H, C_6H_4)
